# Modelling the Impact of Mass Testing to Transition from Pandemic Mitigation to Endemic COVID-19

**DOI:** 10.3390/v14050967

**Published:** 2022-05-05

**Authors:** Joel R Koo, Alex R Cook, Jue Tao Lim, Ken Wei Tan, Borame L Dickens

**Affiliations:** Saw Swee Hock School of Public Health, National University of Singapore, National University Health System, Singapore 117549, Singapore; ephkoor@nus.edu.sg (J.R.K.); juetao@nus.edu.sg (J.T.L.); kenwei@nus.edu.sg (K.W.T.); ephdbsl@nus.edu.sg (B.L.D.)

**Keywords:** SARS-CoV-2, mass testing, rapid antigen test, agent-based model, endemicity

## Abstract

As countries transition from pandemic mitigation to endemic COVID-19, mass testing may blunt the impact on the healthcare system of the liminal wave. We used GeoDEMOS-R, an agent-based model of Singapore’s population with demographic distributions and vaccination status. A 250-day COVID-19 Delta variant model was run at varying maximal rapid antigen test sensitivities and frequencies. Without testing, the number of infections reached 1,021,000 (899,400–1,147,000) at 250 days. When conducting fortnightly and weekly mass routine rapid antigen testing 30 days into the outbreak at a maximal test sensitivity of 0.6, this was reduced by 12.8% (11.3–14.5%) and 25.2% (22.5–28.5%). An increase in maximal test sensitivity of 0.2 results a corresponding reduction of 17.5% (15.5–20.2%) and 34.4% (30.5–39.1%). Within the maximal test sensitivity range of 0.6–0.8, test frequency has a greater impact than maximal test sensitivity with an average reduction of 2.2% in infections for each day removed between tests in comparison to a 0.43% average reduction per 1% increase in test frequency. Our findings highlight that mass testing using rapid diagnostic tests can be used as an effective intervention for countries transitioning from pandemic mitigation to endemic COVID-19.

## 1. Introduction

Since the deployment of mass vaccination and boosting, countries that had effective pandemic mitigation strategies have grappled with how to transition from mitigation to endemic COVID-19. Coupled with the resumption of international travel and the emergence of novel strains, this change in stance has often been accompanied by a “liminal” wave of COVID-19, as the virus spreads in populations that have vaccine-induced, but little infection-inducted, immunity—over the past year this has included Australia, China, New Zealand and Singapore, among others. Surges in cases are expected as vaccines do not fully prevent infection but instead prevent most severe disease [1]. Waning of protection and efficacy against new strains are also of concern [2]. Interventions which are minimally disruptive are therefore desirable as countries segue between mitigation and endemicity.

In this study, we explore the impact of such a nondisruptive measure with the implementation of routine rapid testing (RRT) in a local population. RRTs utilize a rapid test, such as an antigen-rapid diagnostic test (Ag-RDT), which, when compared to the reverse transcription polymerase chain reaction (RT-PCR) test, is cheaper, faster in obtaining a test result and can be self-administered, although test sensitivity is lower [3]. Ag-RDTs can additionally be distributed quickly, made readily available to the general population and provide a means to detect hidden infections including those which are asymptomatic, pre-symptomatic or have very mild symptoms not requiring medical attention [4]. With RRT, surveillance can be expanded beyond high-risk groups, such as healthcare workers or nursing home residents, to members of the general population, allowing them to be isolated, or to self-isolate, upon infection, reducing onward spread. This may be sufficient to blunt the size of the liminal wave, preserving healthcare capacity and the quality of care. 

Here, we examine mass testing through an agent-based model of a city, which is modelled upon the city-state of Singapore during the period of the B.1.617.2 Delta strain outbreak that started in July 2021. To prepare the city to transition into an endemic COVID-19 state, the Singapore government had announced a general policy of vaccinating, tracing and testing of the population on 31 May 2021 [5]. On 27 September 2021, with 81% of the population vaccinated (mostly receiving two doses of mRNA vaccines), daily cases had risen to a level of 1900 that led to Singapore going into a state of semi-lockdown, named the “Phase 2 Heightened Alert (stabilization period)”. The NPIs thus implemented restricted community spread of COVID-19 but due to their broader impact on society were not sustainable. Our study therefore seeks to explore an alternate approach of applying RRT on a national scale, assessing whether it could reduce the epidemic peak during the period of October 2021 to December 2021, negating the need for NPIs, preventing new infections and consequently the burden on critical healthcare resources. Through use of RRT, we estimate the number of COVID-19 cases averted through RRT, the reduction in effective reproduction number $R\left(t\right)$ and number of ICU cases averted.

## 2. Materials and Methods

We modified the geographical, demographic and epidemiological model of Singapore for respiratory diseases (GeoDEMOS-R) [6], an agent-based model for this study. Several adjustments and updates to the model were made for vaccination, agent behavior, case detection through contact tracing and the inclusion of mass testing as an intervention. 

**Figure 1:**
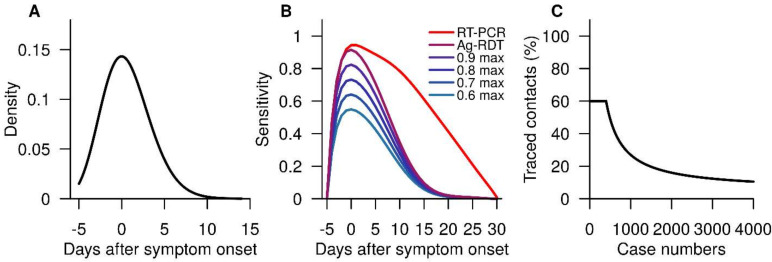
Figures of some parameters used within GeoDEMOS-R. (**A**) Infectiousness of an infected individual relative to the day of symptom onset. (**B**) RT-PCR and Ag-RDT test sensitivity profiles. (**C**) Proportion of traced contacts of detected cases, which diminishes as the cases increase.

The transmission mechanism of the model operates at an individual level [6]. Suppose two individuals within the synthetic population, *i* and *j* are in contact where *j* is infectious, we denote the probability of *j* infecting *i* on day *t* in location type *g* by

(1) $$P_{ijg}=\beta C_{g}S_{i}I\left(t-t_{j}\right),$$

where $\beta \in \left(0,1\right)$ is the infectiousness parameter, $C_{g}$ the contact parameter of location type *g*, $S_{i}$ the susceptibility of *i* and $I\left(t-t_{j}\right)$ the infectiousness of *j* on day *t* (Figure 1a) given that j was infected on day $t_{j}$. In the event j infects i, i will undergo an incubation period, during which i will eventually become infectious and proceed to infect other individuals that i comes into contact with. This occurs daily across the synthetic population among infectious individuals and their contacts.

### 2.1. Vaccination

Individuals within the synthetic population were assigned vaccination status (vaccinated or unvaccinated) according to age-stratified vaccination data from Ministry of Health, Singapore (MOH) [7]. Vaccinated individuals within the simulation were set to be 50% less susceptible to being infected [8] and if infected, their infectiousness was 30% less than an unvaccinated individual [8]. The vaccination proportion utilizes data made available up until 23 August 2021 with individuals within the different age groups selected randomly to receive vaccination.

### 2.2. Agent Behavior

The model assumes that the symptomatic rate of COVID-19 was 60% of infections, including infected individuals who present mild symptoms [9]. Symptom onset followed an incubation period with a gamma distribution [10]. The proportion who would seek medical attention were modelled to have a random delay of 0–4 days after they display symptoms. About 25% of symptomatic individuals under 60 [11], and all symptomatic individuals above the age of 60, are assumed to seek medical attention. The remaining proportion of symptomatic individuals are assumed to isolate themselves at home. A PCR test will be administered to those who seek medical attention. An infected individual will test positive based on PCR sensitivity [12], which is relative to the day of symptom onset.

### 2.3. Contact Tracing

Upon confirmation of a COVID-19 case, contact tracing of familial and community contacts is carried out. Familial contacts of confirmed cases will begin quarantine for a duration of 14 days. They will also undergo two rounds of PCR testing, one on the first day of quarantine, and another a week later; this was based on the prevailing public health measures in Singapore at the time. Contact tracing commences on the contacts of those under quarantine and familial members who become positive. Community contacts were tracked through a simulated version of the national contact tracing system TraceTogether [13]. We modelled the tracing capabilities of the contact tracing system as follows: For the first 400 confirmed individuals per day [14], their community contacts are traced over a period of 5 days, up to a maximum of 60% of contacts. The proportion of traced contacts is assumed to diminish subsequently to a minimum level of 5% (Figure 1c) as the number of infections increase, reflecting burdens on the contact tracing system. Traced community contacts are tested and placed under quarantine for 14 days, starting from the time when they were informed about their exposure.

### 2.4. Baseline Model

We obtained the total number of COVID-19 infections and reported cases which consist of individuals who have tested positive via a PCR test. Fitting was then performed using daily observed COVID-19 case data in Singapore for the period of 23 August 2021 to 8 October 2021, comprising of 46 days [15]. We selected data from this time frame as it represented a period which consisted of observations pertaining to the initial phase of an outbreak of COVID-19 (Delta strain) in the Singapore population, during which time more relaxed community measures were in place. We first fit the data to an exponential model $\exp\left(rt+b_{0}\right)$, where r represents the initial growth rate of the epidemic and obtain a distribution of r values. The relationship between r and β, the infectiousness parameter, was determined from our previous work [16], which was utilized to derive a β value from a sampled value of r. We sampled 200 values of r and used the corresponding β values as the infectiousness parameters for our baseline simulations.

### 2.5. Mass Testing Intervention

As other mitigation measures were minimal and kept constant through the time period, all other parameters aside from those involved in the Ag-RDT testing intervention were kept constant. This allowed for the measurement of RRT’s effects through measuring the deviation from the baseline in three specific areas, namely the changes in overall size of the outbreak, the number of cases requiring intensive medical care (ICU cases) as well as the effective (instantaneous) reproduction number $R\left(t\right)$. To measure the effect of mass testing on ICU cases, we fitted an autoregressive distributed lag model using ICU and observed cases. The model was applied to the simulation outputs to estimate the expected ICU numbers in each scenario.

By using an individual-based model, we have complete records of the transmission events within simulations, in particular the time between infections, i.e., generation times for infectious individuals. Therefore, we were able to compute the modelled $R\left(t\right)\;$ [17] using the outputs given from our simulations as

(2) $$R\left(t\right)=\frac{I_{t}}{\sum_{n=0}^{t-1}w_{n}I_{n}}\;,$$

where $I_{t}$ is the number of individuals infected on day t and w is the generation time distribution such that $\sum_{n=0}^{t-1}w_{n}=1\;$

During an intervention run, at the point of implementing the mass testing, the synthetic population was partitioned into Ag-DRT testing groups; each group underwent mandatory testing on specific days. On the assigned day of an individual’s test, the individual will self-administer an Ag-RDT with a sensitivity profile (Figure 1b). We modelled the healthcare-seeking behavior to be similar to that of a symptomatic individual, with 25% of those tested positive from Ag-RDT seeking medical attention and undergoing further confirmation through a PCR test, and the remaining 75% undergoing self-isolation at home.

We considered scenarios with two distinct implementation time points. The first scenario began implementing mass testing 30 days into the simulations and the other at the peak of the modelled outbreak. The implementation time points of 30 days and the outbreak peak are referred to as “early” and “peak” implementation of mass testing, respectively. For each of these implementation times, we also varied the mass testing frequency with fortnightly, weekly and tridaily (once every 3 days) testing. We ran 200 simulations for each scenario using various combinations of implementation time point, test frequency and maximal test sensitivity with a run length of 250 days for all simulations designed to begin from 23 August 2021. 

We also estimated the reduction in infections from baseline by taking the simulation outputs from the current set of intervention parameters and fitting it to a generalized additive model.

## 3. Results

### 3.1. Baseline Scenario

**Figure 2:**
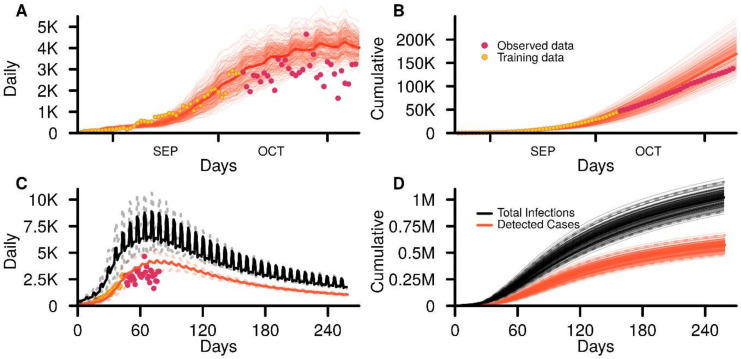
GeoDEMOS-R baseline outputs from 200 simulations. (**A**) Daily and (**B**) cumulative detected cases for the initial 90 days of baseline simulations. (**C**) Daily and (**D**) cumulative infections and detected cases for the entire 250 days of baseline simulations. Dashed lines represent the 95% interval.

The baseline simulations showed that there were 1,021,000 (899,400–1,147,000) COVID-19 infections in the resident population at the end of 250 days of which 571,000 (493,000–654,000) infections were detected as cases due to clinic-based testing or through contact tracing (Figure 2). The number of detected cases represent 55.9% of total infections. At the height of the outbreak, the number of daily infections reached 8930 (7390–10,600) and occurred 71 (64–78) days after the start of the simulations. The peak in the daily detected cases occurred 6 days after the infection peak on day 77 (69–89) with 4380 (3580–5320) detected cases or 49.0% of that of the peak daily infection number.

### 3.2. Mass Testing with Ag-RDT

With a maximal test sensitivity of 0.6 and early RRT intervention, fortnightly testing resulted in 890,000 (776,000–1,010,000) infections. The intervention resulted in 130,000 (121,000–140,000) averted infections, representing a decrease of 12.8% (11.3–14.5%) in total infections of the baseline scenario. When the implementation time point was delayed to the peak of the outbreak, the number of infections increased to 909,000 (791,000–1,040,000). The mitigating effect of implementation at the peak was, however, similar to the early implementation setting with 111,000 (103,000–120,000) infections averted and a decrease of 11.0% (9.51–12.6%) in total infections from the baseline. 

Increasing the testing frequency to weekly or tridaily resulted in a proportional reduction in infection numbers. Weekly RRT implemented at the early and peak time point resulted in 764,000 (646,000–885,000) and 802,000 (686,000–927,000) infections, with 256,000 (247,000–268,000) and 219,000 (206,000–227,000) cases being averted or 25.2% (22.5–28.5%) and 21.5% (19.0–24.9%) of infections averted, respectively. For tridaily testing, the number of infections for the early and peak implementation were 470,000 (353,000–590,000) and 582,000 (468,000–698,000), with infections averted at 551,000 (536,000–564,000) and 424,000 (406,000–443,000), or 54.1% (48.2–60.7%) and 43.1% (38.4–47.9%), respectively. The summary of the changes to the baseline in a scenario with improved maximal test sensitivity of 0.8 can be found in Table 1.

**Figure 3:**
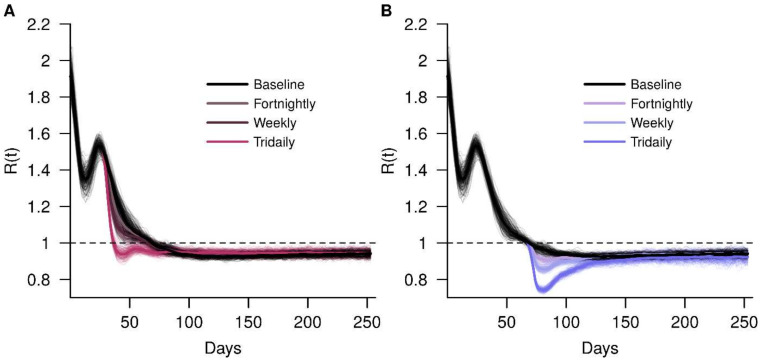
Effects of RRT on the COVID-19 transmission by measuring the effective reproduction number $R\left(t\right)$ of the baseline simulation, fortnightly, weekly and tridaily; RRT interventions implemented at (**A**) early implementation and (**B**) peak implementation.

The $R\left(t\right)$ was 1.9 at the beginning of all simulations. In the baseline scenario, $R\left(t\right)$ decreased to 1 on day 71 and remained below 1 for the remainder of the simulation. For early implementation and a maximal test sensitivity of 0.6, tridaily RRT brought the $R\left(t\right)$ below 130 days sooner (Figure 3a), corresponding to a reduction of 54.1% in cases. Peak RRT implementation affected $R\left(t\right)$ in a different manner. Since the $R\left(t\right)$ was close to 1 when the intervention occurred, increasing the test frequency resulted in a larger deflection of the $R\left(t\right)$ away from 1, to a minimum of 0.74 under tridaily testing (Figure 3b). The number of infections averted is fewer, however, when compared to early implementation as there are fewer testing events in the former.

**Figure 4:**
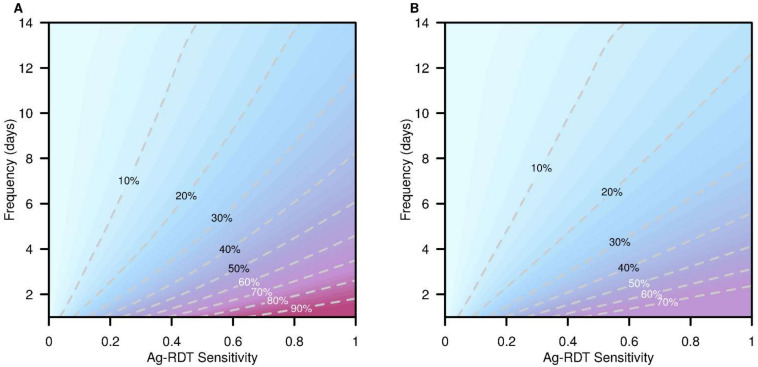
Percentage reduction in COVID-19 infections with various RRTs. Infection reduction values derived from the generalized additive model, with reductions at various test frequencies and maximal Ag-RDT sensitivities. (**A**) Infection reductions from the baseline with RRT at early implementation. (**B**) Infection reductions from the baseline with RRT at peak implementation.

The trends in percentage reduction of infections across different test sensitivities and frequencies are presented in Figure 4. We see diminishing levels of infection reduction when delaying the implementation of RRT as more infections have already taken place. This was shown with the rotation of the level curves about the bottom left corner of the parameter grid in a clockwise fashion. With early implementation alongside very high test sensitivity and frequency, the percentage of averted infections can reach 94.5% whereas the maximum percentage of cases averted for peak implementation was 71.2%.

Within the maximal test sensitivity range of 0.6–0.8 and with early implementation, test frequency has a greater impact than maximal test sensitivity with an average reduction of 2.2% in infections for each day removed between tests, in comparison to a 0.43% average reduction per 1% increase in test frequency.

### 3.3. ICU Cases

**Figure 5:**
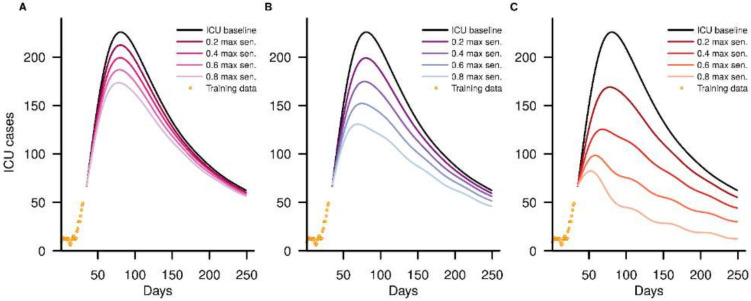
Baseline ICU cases and dampening of ICU case trajectories with RRT intervention at early implementation at various maximal test sensitivity (0.2, 0.4, 0.6, 0.8), with (**A**) fortnightly testing, (**B**) weekly testing and (**C**) tridaily testing.

The autoregressive distributed lag model of ICU cases showed that in our baseline scenario, ICU utilization would peak at 226 (185–271) cases. When early RRT was implemented, the primary effect on the ICU trajectory was to reduce the peak size, an effect that was more marked with increased maximal test sensitivity and frequency. Fortnightly testing had little effect, even at a high maximal test sensitivity of 0.8, reducing the peak utilization by 22.9% (Figure 5a). For weekly testing (Figure 5b), a peak reduction of at least 32.6% was observed when the maximal test sensitivity was more than 0.6. When increasing the frequency further to tridaily, we observe flattening of the ICU case curves (Figure 5d), with a maximum of 99 (64–139) ICU cases, or 56.4% of the reduction of the peak size, at a maximal test sensitivity of 0.6.

## 4. Discussion

Our findings suggest that the magnitude and impact on society and the healthcare system of a post-mitigation liminal wave of COVID-19 can be lessened by the mass use of Ag-RDT RRT. Where the maximal test sensitivity is less than ideal (0.6 maximal test sensitivity), our simulations showed that the reduction in infections, depending on the time of intervention implementation and test frequency, ranged from 11.0–54.1% when compared to the baseline scenario. Only a tridaily (once every 3 days) test frequency implemented early in the outbreak resulted in an extended period of $R\left(t\right)$ being less than 1 for 30 days and led to the greatest infection reduction of 54.1% compared to baseline. The ICU case reduction for this intervention configuration was also prominent with a reduction of 56.4% compared to the baseline ICU peak of 225 ICU cases. The findings demonstrate the protection that mass testing could confer, even if the utilized tests had a lower sensitivity than the PCR tests. Regular mass testing of the population is therefore a possible strategy that can be employed to effectively stem the spread of a COVID-19 outbreak.

Our estimates on the mitigating effects of mass testing highlights its potential use as a nondisruptive intervention during an outbreak as it is able to reduce up to half of all infections and peak ICU cases even at a low maximal test sensitivity. RRT can differentiate infected and susceptible individuals en masse and prevent the unnecessary quarantining of large populations over long durations. Combined with the relatively quick test turnaround, mass testing ensures that individuals are regularly informed of their current infection status, and thus are able to determine if they should proceed with their regular daily routine or self-isolate or quarantine without the need of waiting for authorities to enforce isolation or quarantine measures. As highly infectious COVID-19 variants exist with short incubation times, outbreaks can have rapidly increasing case numbers which require highly restrictive measures to be aggressively implemented. By employing Ag-RDT RRT, greater targeting would capture more infected individuals and prevent high peak outbreaks by isolating more individuals and preventing them from interacting with others in the wider community. 

In the face of a rising epidemic, lockdowns can reduce the spread of COVID-19 in a population by slowing the spread of disease through minimizing contact events. However, its success, economic and societal costs, grow proportionally with duration and severity where the negative effects can rapidly eclipse the derived benefits as the population and economy’s wellbeing deteriorates. Concerns additionally exist with implementations and exit strategies from a prolonged lockdown as factors including implementation timing and the rate at which the measure ease [16,18] play a crucial role in limiting undesired outcomes such as a rebounding epidemic or the need for a secondary wave of lockdown measures. 

However, the success of RRT is dependent on compliance of members of the public. Should individuals choose not to self-isolate or quarantine when they are identified as positive or suspected of being exposed, other measures will be required. Additional interventions including workplace, school or spot testing may be required to culminate in an environment of routine testing in an endemic COVID-19 state. Institutional isolation or quarantine can be utilized for individuals who are not compliant and require additional surveillance [19] but the majority should be able to quarantine or isolate at their place of residence, although in some situations where high-risk individuals such as the unvaccinated elderly reside there, the option to isolate or quarantine elsewhere could prevent adverse health outcomes. Being able to recover or reside in an individual’s place of residence is appealing to countries with high vaccination rates where severe outcomes from COVID-19 are severely reduced [20] as it lowers healthcare burdens to both the individual and government, and promotes better overall population wellbeing. The use of mobile technologies [21,22] and at-home testing also reduces contact rates of infected and exposed individuals, and can be regularly updated with remote advice and instructions able to be given through telehealth. Overall, adapting to an institutional–home-based hybrid recovery system for the population, targeting those who are at high risk of poor health outcomes for intensive monitoring, can support healthcare infrastructure recovery.

Limitations of this study include assumptions of agent behavior in the model in terms of adherence to distancing measures and health-seeking behavior. These behaviors were assumed to be constant but we acknowledge that behavioral dynamics of individuals may change as the epidemic progresses, affecting its trajectory. Behavioral changes may include falling usage of Ag-RDT and compliance to other NPIs in place during the liminal phase, such as group size restrictions or other social restrictions. Further NPIs may be implemented over time which may also affect RRTs in terms of identified cases although we expect the efficacy to remain the same in terms of capturing the same proportion of cases according to the set maximal test sensitivity and frequency. Vaccination parameters may affect RRTs in terms of waning and efficacy versus newer strains. Newer variants may also have differing incubation times and infectious periods, which will require further parameterization of the model. Lastly, differences in parameters such as symptomatic rate, compliance to self-isolation, the proportion seeking medical attention and delays in the publication of test results may cause the testing strategies to be more or less effective depending on the medical infrastructure and behaviors of a population under study.

Nevertheless, our findings suggest that countries yet to transition fully to an endemic state may consider using mass testing to ameliorate the impact of relaxing mitigation measures.

## Figures and Tables

**Table 1:** Infections and reduction relative to baseline scenario at 0.8 maximal test sensitivity.

		Maximal Sensitivity							
		0.6				0.8			
**Testing**	**Baseline**	**Early Implementation**		**Peak Implementation**		**Early Implementation**		**Peak Implementation**	
**Frequency**		**Infections**	**Reduction (%)**	**Infections**	**Reduction (%)**	**Infections**	**Reduction (%)**	**Infections**	**Reduction (%)**
Fortnightly	1,021,000 (899,400–1,147,000)	890,000	12.8	909,000	11.0	843,000	17.5	871,000	14.7
		(776,000–1,010,000)	(11.3–14.5)	(791,000–1,040,000)	(9.51–12.6)	(727,000–969,000)	(15.5–20.2)	(752,000–998,000)	(12.9–16.7)
Weekly		764,000	25.2	802,000	21.5	671,000	34.4	732,000	28.4
		(646,000–885,000)	(22.5–28.5)	(686,000–927,000)	(19.0–24.9)	(557,000–794,000)	(30.5–39.1)	(616,000–854,000)	(25.4–32.1)
Tridaily		470,000	54.1	582,000	43.1	298,000	71.1	476,000	53.5
		(353,000–590,000)	(48.2–60.7)	(468,000–698,000)	(38.4–47.9)	(196,000–402,000)	(64.2–78.4)	(376,000–581,000)	(49.2–58.3)

## Data Availability

The data used within this study can be made available upon request.
